# Health Tract, No. 172

**Published:** 1865

**Authors:** 


### HEALTH TRACT, No. 172.

### LOGIC RUN MAD.

"What is good for the goose is good for the gander," may have a certain amount of truth in it; but what is good for a goose is not necessarily, and therefore, good for a jackass. Yet this is the line of argument used by many, and is sometimes found in books, and magazines, and newspapers, in reference to health and disease. A man, for example, is sick of any thing or nothing; takes something and soon gets well; he has great faith in that medicine, and thereafter takes it for every ailment in his own person, and recommends it freely and confidently to any one who may be sick, without any special regard to the nature of the malady.

Another man makes brandy and water, especially the brandy, a panacea for all his ails, and recommends it as a useful and efficient medicine to any friend who may happen to complain, whether it be of belly-ache, bilious colic, or cancer.

It has been stated many times in print, that the Russians give their infants a warm bath, and, even when newly born, roll them out in the snow, and therefore it must be a good practice for all children. But are Russian children, as to the masses, unusually thrifty? According to one of their late publications, the *Rousky Dnevnik*, the mortality is such as to force public inquiry as to its cause; whether by or in spite of snow-baths, we say nothing.

The working-out mothers in Dresden bandage their children in the morning so completely that they can do nothing but roll over and over, and thus they remain all day, with a feeding at noon, thereby saving the expense of a nurse, and keeping them out of mischief. But shall we therefore follow the example of Dresden mothers, and keep our children helplessly bandaged the whole day, they meanwhile sweltering in all their excrements? Is it any wonder that one child out of ten born in Dresden is deformed? The greater wonder is, that nine out of ten children thus treated do not die outright.

We hear a great deal, in water-cure journals and others, of the thoroughness and efficiency of Turkish baths. If this is among the lower orders, then there is not a dirtier race in existence; if among the higher classes, we know they are not excelled for their effeminacy and early mortality. Because the masses of Chinese live mainly on rice, and the Irish on potatoes, vegetarians would persuade us that mankind would live longer if no meat were eaten. The Chinese are vegetarians perforce, and as a nation are the most filthy, beastly, effeminate people on the globe; and as for the race which lives almost exclusively on potatoes, are they exceeded by any people on this planet in diminished mental calibre, ignorance, low cunning, black-hearted revenge, and bestiality in strong drink? Where is the housekeeper who is not conscious that at least as to the menial race there is no truthfulness, no honesty, but in their place a fawning deceitfulness, unendurable by generous minds? And gymnasts run mad in their laudations of the games and sports described in all Greek and Roman story, and yet when these nations were at the very height of their civilization, they were most vilely corrupt, degenerate, and debased. The only efficient system of gymnastics is steady, useful, and remunerative labor. In short, before we adopt any means of health aside from temperance and industry, let us first ascertain certainly that others have been wholly benefited, and not all injured thereby; otherwise we are but putting in practice a "mad logic."

